# Acculturation orientations mediate the link between religious identity and adjustment of Turkish-Bulgarian and Turkish-German adolescents

**DOI:** 10.1186/s40064-016-2688-1

**Published:** 2016-07-08

**Authors:** Radosveta Dimitrova, Arzu Aydinli-Karakulak

**Affiliations:** Department of Psychology, Stockholm University, Frescati Hagv. 14, 106 91 Stockholm, Sweden; Department of Psychology, Hiroshima University, Kagamiyama 1-3-2, Higashihiroshima City, Hiroshima 739-8511 Japan; Department of Psychology, Bahçeşehir University, Çırağan cad. Osmanpaşa mektebi sok. 4/6, Beşiktaş, Istanbul, Turkey

**Keywords:** Religious identity, Acculturation orientations, Socio-cultural adjustment, Turkish-Bulgarian and Turkish-German youth

## Abstract

There is a growing recognition of the need to examine religiousness and conduct research on its influence on acculturation and adjustment among ethnic minorities (Güngör et al. in Int J Behav Dev 36:367–373, 2012. doi:10.1177/0165025412448357). The present study compares Turkish minority youth in Bulgaria and Germany by examining relationships among religious identity, acculturation orientations (i.e., cultural maintenance and adoption) and acculturation outcomes (i.e., life satisfaction and socio-cultural adjustment to the Turkish and mainstream cultures). Participants were 161 youth in Bulgaria and 155 in Germany who completed measures on religious identity, acculturation orientations and adjustment. Results revealed that religious identity and Turkish culture maintenance are more important for Turkish-German, than for Turkish-Bulgarian youth. A multigroup path model showed that for both samples acculturation orientations partially mediated the link between religious identity and adjustment to the Turkish culture, whereas religious identity was directly related both to adjustment to the mainstream culture and to life satisfaction. Findings highlight the centrality of religious identity and Turkish domains of acculturation for positive adjustment outcomes for Turkish youth in Bulgaria and Germany.

## Background

There is a growing recognition of the need to examine religiousness and conduct research on its influence on acculturation and adjustment among ethnic minority youth (Güngör et al. 2012). Examining religious identity and acculturation orientations during this developmental stage is particularly important because adolescents often belong to multiple social groups and the process of forming a social identity represents a crucial developmental task for them (Kroger and Marcia 2011). This process becomes particularly salient for ethnic minority adolescents who have to navigate among multiple social groups and cultures (Benet-Martínez and Haritatos 2005; LaFromboise et al. 1993). It is during this stage that youth acquire a deeper understanding of their social identities (including religious identity) and their orientation towards cultural maintenance and adoption.

In fact, strong religiousness is associated with enhanced adjustment of individuals in general and ethnic minority groups in particular (Friedman and Saroglou 2010). This implication fuels an increasing interest in studying the role of ethnicity and culture in relationship with religiousness, acculturation and adjustment. However, there is a dearth of research examining correlates of acculturation orientations and their relationships across ethnically diverse samples, and in Turkish ethnic minority living in Eastern Europe in particular. A focus on Eastern Europe and minority groups in this part of Europe is relevant in light of recent political and socio-cultural transformations with significant influence on identity and acculturation of these groups. A unique feature of Turkish-Bulgarians regards the changing cultural and political context in which they live—the collapse of communism and a need to restore a sense of identity as a basis to negotiate the relations between different ethnic groups in Eastern Europe. These contextual features are relevant in affecting identity and acculturation processes of youth. Therefore, we set up to take advantage of this unique contextual feature in exploring beneficial or protective effects of religious identity for Turkish-Bulgarian youth. For that reason, the current study was designed to assess the influence of religious identity and acculturation orientations on adjustment among Turkish-Bulgarians compared to Turkish-Germans. We specifically focus on Turkish groups in Bulgaria and Germany because even though these two groups share certain characteristics, they also represent two very distinct contexts of settlement. Comparing acculturation conditions, acculturation orientations and acculturation outcomes and the relationships between these components in two diverse settings sheds light on how adolescents’ acculturation process is affected by cultural context by delineating the commonalities and differences in both samples.

### Acculturation framework

We apply the acculturation framework proposed by Arends-Tóth and van de Vijver (2006) that differentiates acculturation conditions, orientations, and outcomes. Acculturation describes patterns of cultural and psychological changes that result from the integration of individuals from two or more cultures, which often result in long-term psychological and socio-cultural adaptations between both groups (Berry et al. 2006; Berry 2003). Acculturation conditions refer to personal characteristics (family, personality, identity, specifics of minority groups), and to characteristics of the receiving and host society such as discrimination and opportunity structures. Acculturation orientations refer to the maintenance of one’s culture of origin and the extent to which minority groups actively participate in the mainstream culture. Acculturation outcomes concern the degree of success of the acculturation process in terms of psychological adjustment as well as socio-cultural adjustment in both ethnic and host cultures.

In this study, we focus on Turkish youth in Germany and Bulgaria and operationalize their acculturation conditions as *religious identity* (representing feeling of attachment and belonging to own religious group), their acculturation orientations as host culture *adoption* and heritage culture *maintenance*, and their acculturation outcomes as *adjustment to host and heritage cultures* and *psychological adjustment* outcomes (i.e., life satisfaction).

### The acculturation context

Research and theory suggest that macro-level variables filtrate through more proximate circumstances in which children and adolescents grow up (Bronfenbrenner 1979). In case of the acculturation process, a similar stance has been put forward by Bourhis et al. (1997) in their Interactive Acculturation Model (IAM). The IAM claims an interactionist relationship between the individual and the acculturative context: State policies and other circumstantial conditions affect individuals’ acculturation orientations and also the outcomes that emerge from different combinations of contextual conditions and individual-level acculturation orientations. Hence, when examining the process of acculturation, its conditions, orientations and outcomes, it seems inevitable to take contextual variables into account.

The notion that contextual conditions affect the acculturative process of individuals has also been supported by empirical findings. In a four country comparison of Turkish groups, evidence has shown that minority groups in the country with the most assimilation pressure (France) report high scores on sociocultural adjustment to the mainstream culture, combined with low scores on psychological adjustment compared to minority groups of countries with less assimilation pressure (Australia, the Netherlands, and Germany) (Yagmur and Van de Vijver 2012). Further support for the context-specificity in acculturation is reported by Vedder and Virta (2005) with Turkish groups in Sweden and the Netherlands. The authors found strong culture maintenance in the Swedish sample and strong language assimilation in the Dutch sample, but weak support for integration (adopting both heritage and host cultures) in the Swedish sample. In summary, findings suggest that the acculturation process varies across social contexts due to tangible opportunities for (Turkish) minority groups in diverse acculturative contexts.

### Turkish minority in Bulgaria versus Germany

Based on theoretical and empirical evidence, we consider essential to study and compare the acculturation process of youth in light of their contextual backgrounds. We chose to focus on Turkish groups in Bulgaria and Germany, because even though these two groups share certain characteristics, they also represent two very distinct contexts of settlement. First, both groups are similar as they represent the largest minority group in terms of size and prominence (10 % of about 8 million Bulgarians; close to 4 % of the 80 million German population) with a separate culture, language, and religion (National Statistical Institute 2004; Migration-report commissioned by the German Federal Office for Migration and Refugees 2013). They are a predominantly Muslim group in a country where Christian Orthodoxy (Bulgaria) and Catholicism and Protestantism (Germany) is the official religion (Eminov 2007). In both countries, Turkish groups share a strong supportive network, form cohesive ethnic communities, and maintain their cultural adherence to Turkish traditions.

Second, these two groups also differ. The Turkish minority in Bulgaria has been in the country for centuries unlike Turkish labor migration to receiving countries such as Germany that has happened more recently in the 1960s. Moreover, the Turkish-Bulgarian community inhabits disadvantaged areas characterized by high unemployment rates, poor infrastructure, and low professional opportunities compared to the average country levels (Maeva 2005). Finally, the most distinctive characteristic associated with this minority regards the assimilation policy of the Bulgarian government in the late 1980s. The government conducted several renaming campaigns, representing one of the most rapid assimilation campaigns in European history, coercing nearly one million people to change their names (Dimitrov 2000). Hence, Turkish-Bulgarians are characterized by severe historic assimilation experiences and persistent social disadvantages. Turkish-Germans, on the other hand, were exposed to relatively more favorable acculturation conditions (e.g., targeted policy across areas of education, labor, and urban development, and reforms for a better integration and managed migration; Bendel 2014) compared to the heavily oppressed Turkish-Bulgarians.

### Acculturation condition: religious identity

Religious identity, operationalized here as an individual’s sense of belonging and commitment to a religion (Nesbitt and Arweck 2010), is part of the acculturation process. Following past research, we utilize identity as a proxy for acculturation condition, because it reflects a basic personal characteristic as specified in the model guiding our study (Arends-Tóth and van de Vijver 2006) as well as determines coping capacities and psychological adjustment outcomes (Hofer et al. 2006). Across a variety of cultural contexts, it has been documented that religious identity is an important asset for ethnic minority adolescents, regardless of their specific religious affiliation (Lopez et al. 2011; Wallace et al. 2003). Ethnic minority adolescents have been found to score high on religious identity as well as on religious participation and to assign great importance to religion and prayer (Harker 2001; Verkuyten et al. 2012).

Additionally, religious identity has been documented as particularly salient for members of Muslim minority communities (Verkuyten and Yildiz 2009). This pattern has been confirmed across different European countries for both youth and emerging adults (Haddad and Smith 2001; Phalet 2004). Findings suggest that religious identity is likely to be strong when members of minority groups feel a strong affiliation to their ethnic community, which might also result from anti-religion policies (Dimitrova et al. 2014a). Across studies, Turkish youth have been found to demonstrate higher religiosity and an overall stronger religious identity as important source of identification and adjustment compared to mainstream youth (Dimitrova et al. 2014b). These findings are consistent with research documenting that Muslim youth in general display a more pronounced religious identity than their non-Muslim peers (Saroglou and Galand 2004; Saroglou and Mathijsen 2007).

In terms of correlates of religious identity, findings seem mixed. While there is some research that underlines religious identity’ beneficial effects, there is also research on how religious identity might turn into an obstacle for acculturating adolescents. A strong religious identity has been found to be associated with low levels of delinquency (Junger and Polder 1993), disruptive behaviors (Abbotts et al. 2004; Udel et al. 2011), and internalizing and externalizing problems (Bartowski et al. 2008). On the other hand, however, we have evidence showing that more religious Muslims are less willing to adopt the mainstream culture (Friedman and Saroglou 2010; Güngör et al. 2011; Saroglou and Mathijsen 2007) which in turn increases the social and cultural distance between Muslim minorities and mainstream society.

### Acculturation orientations: maintenance and adoption among Turkish minority youth

Much research has been devoted to study how acculturation orientations may relate to adjustment outcomes. With few exceptions, maintaining one’s original culture, while at the same time adopting the host culture (termed integration, Berry 1997) seems to be the most adaptive acculturation strategy (Berry et al. 2006; Sam and Berry 2006). However, there is scarce work examining the process of acculturation among Turkish youth in Eastern Europe. One study we are aware of investigated the association between ethnic identity (heritage and mainstream), acculturation orientations (host culture adoption and heritage culture maintenance) and psychological and sociocultural outcomes in Turkish-Bulgarian and Turkish-German youth (Dimitrova et al. 2015). The authors found that youth in both cultural contexts regarded their Turkish culture maintenance as more important than host culture adoption. Turkish-Bulgarians also reported higher scores on host culture adoption than Turkish-Germans. An important finding was that Turkish maintenance was positively related to adjustment to both cultures, whereas mainstream adoption was positively associated with adjustment to the host culture only. The authors further suggest that acculturation orientations exemplified by Turkish maintenance are salient factors for adjustment of Turkish minority adolescents from two different European countries. Relatedly, results of another study support the association between acculturation orientations and adjustment for Turkish adults living in Bulgaria and the Netherlands, while also revealing substantial differences in salience and relations among cultural maintenance, adoption and adjustment between these two groups (Dimitrova et al. 2014b). Turkish-Bulgarians reported higher host culture adoption than the Turkish-Dutch, whereas the opposite pattern emerged for Turkish culture maintenance. As expected, Turkish adults in the Netherlands showed a more pronounced tendency to maintain their heritage Turkish culture compared to their Turkish counterparts in Bulgaria.

Overall, findings seem to be indicative of differences in salience of acculturation orientations among Turkish-Bulgarian and Turkish-German youth. Moreover, findings also seem supportive for the acculturation framework proposed by Arends-Tóth and van de Vijver (2006) confirming strong relationships between acculturation orientations and adjustment outcomes.

### The present study

**Fig. 1:**
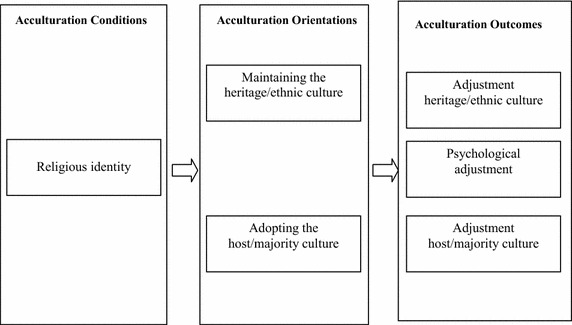
Conceptual model of acculturation conditions, orientations and outcomes of Turkish youth

We follow this line of research and address questions about both mean level and structural similarities and differences in Turkish-German and Turkish-Bulgarian adolescents’ acculturation process. We assume that acculturation conditions (religious identity) influence acculturation outcomes (socio-cultural and psychological adjustment) and that this relationship is mediated by the acculturation orientations (maintenance of the heritage culture and adoption of the host culture) of Turkish-Bulgarian and Turkish-German minority youth (see Fig. 1 for an overview).

Two research questions were addressed: (1) Do Turkish youth in Germany and Bulgaria differ in their religious identity endorsement and in their acculturation orientations of cultural maintenance and cultural adoption? (2) How are the relationships among religious identity, acculturation orientations and socio-cultural and psychological adjustment in the two groups? First, we expect differences in religious identity endorsement and acculturation orientations among the two youth samples, since the Turkish-Bulgarians belong to a severely stigmatized ethnic minority group in a post-communist atheist country (Ganev 2004; Verkuyten 2005). More specifically, and in line with previous research showing that Turkish-Bulgarians are more likely to report strong attachment to their host Bulgarian culture (Dimitrova et al. 2014a, b), we propose that Turkish-Bulgarians will report lower levels of religious identity and maintenance, and higher levels of adoption than Turkish-Germans. Second, based on the postulated relationship among acculturation conditions, orientations and outcomes in our model of reference (Arends-Tóth and van de Vijver 2006), we test the relationship between religious identity and socio-cultural and psychological adjustment among the two groups. More specifically, we expect that religious identity (i.e., acculturation condition) is positively related to acculturation outcomes and that this relationship is mediated by acculturation orientations of maintenance and adoption in both Turkish groups (for an overview see Fig. 1).

## Method

### Participants and procedure

A total of 313 youth were recruited for this study. The analyses were carried out on a sample of 160 Turkish-Bulgarians with a mean age of 16.44 years (*SD* = .89) and 153 Turkish-Germans with a mean age of 15.40 years (*SD* = 1.45) (see Table 1). With regards to the generational status of our participants, 97 % of the Turkish-Bulgarian youth were born in their host country compared to 94 % of Turkish-Germans. Ninety-four percent of the Turkish-Bulgarians had at least one grandparent that was born in the host country, compared to only 4 % of the Turkish-German youth. Participants came from major towns in Bulgaria and Germany with a high concentration of Turkish-Bulgarian inhabitants: Kardjali, Krumovgrad, and Haskovo; Turkish-German inhabitants: Stuttgart. Prior to data collection, local authorities and schools were informed about the purpose and methods of the study to assure their participation. The sample was recruited through schools with the help of bilingual research assistants. Participants were offered a small gift upon completion of the study. All measures were translated from English into German and Bulgarian by five bilingual speakers adhering to standard guidelines to ensure linguistic equivalence (van de Vijver and Leung 1997). Measures were presented only in Bulgarian and German, because Turkish youth in both countries acquire literacy skills exclusively in Bulgarian/German (Rudin and Eminov 1993). All measures were previously applied with Turkish minority groups in the Netherlands, Germany and Bulgaria, showing excellent psychometric properties in terms of reliability and measurement equivalence in these groups (Arends-Tόth and Van de Vijver 2004; Aydinli and Dimitrova 2015; Dimitrova et al. 2015; Galchenko and Van de Vijver 2007).

**Table 1:** Means and standard deviations of the sample by ethnic group

	Turkish-Bulgarian (*n* = 160)	Turkish-German (*n* = 153)
Age		
Range	15–18	13–19
Mean (SD)	16.44 (.89)	15.40 (1.45)
Gender (%)		
Boys	58	52
Girls	42	48

### Measures

#### Demographic characteristics

Information about participant’s age, gender, place of birth, ethnicity, nationality, and SES (parental occupation and highest level of education) was collected.

#### Acculturation conditions: religious identity

A measure to investigate religious identity was applied following prior work in ethnic minority and Turkish samples (Dimitrova et al. 2014a, b, 2015). The Religious Identity Scale was composed by 21 items such as “I see myself as a member of my religious community”, “I am proud to be a member of my religious community”, and “I have spent much time exploring my religious group (e.g., its rituals, history and traditions)” with internal consistencies of α = .87 for Turkish-Bulgarian and α = .97 for Turkish-German.

#### Acculturation orientations: adoption and maintenance

A scale to measure acculturation orientations was used to capture Turkish and Bulgarian/German language skills, familiarity with social events, as well as the degree of interaction with and knowledge of both cultures. This measure was adapted from previously developed acculturation orientation measure for Turkish minority groups using a two-statement measurement method (measuring attitudes towards both heritage culture and country of settlement (see Arends-Tόth and Van de Vijver 2004; Galchenko and van de Vijver 2007). The Turkish orientation was measured by 13 items such as “I live according to Turkish culture”, “I have Turkish friends”, “I feel Turkish”, and “I celebrate Turkish holidays” (α = .91 Turkish-Bulgarian, α = .93 Turkish-German). Attitudes toward Bulgarian/German culture were measured with 13 items such as “I live according to Bulgarian/German culture”, “I have Bulgarian/German friends”, “I feel Bulgarian”, and “I celebrate Bulgarian/German holidays (α = .81 Turkish-Bulgarian, α = .83 Turkish-German). Items were scored on a 5-point scale ranging from 1 = *strongly disagree* to 5 = *strongly agree* with higher scores indicating higher heritage culture maintenance and adoption, respectively.

#### Acculturation outcomes: socio-cultural and psychological adjustment

A scale to measure sociocultural outcomes in both heritage and mainstream culture was used based on prior work with Turkish samples (Dimitrova et al. 2014a, b). The scale was previously used to measure sociocultural outcomes of Turkish minority groups (see Galchenko and Van de Vijver 2007). This set of items measures sociocultural adjustment outcomes defined as cultural learning processes for successful participation in the host society (Searle and Ward 1990), whereas the acculturation orientation items capture attitudes towards both Turkish heritage and host Bulgarian/German cultures (see Arends-Tόth and Van de Vijver 2004; Galchenko and Van de Vijver 2007). The scale consisted of 36 items about both the Turkish and mainstream cultures (18 items each), again using two-statement format. Participants were asked to indicate the degree of difficulty they experience in daily situations, using a 5-point scale ranging from 1 (*very difficult*) to 5 (*very easy*). Sample items included “Asking advice of Turkish [Bulgarian/German] friends”, “Reading books in Turkish [Bulgarian/German]”, “Making yourself understood by Turkish [Bulgarian/German] friends”, and “Eating Turkish [Bulgarian/German] food” with internal consistencies ranging from α = .91 to α = .87 across samples. Higher scores indicate higher levels of socio-cultural adjustment to heritage and host cultures, respectively.

Additionally, we measured psychological adjustment outcomes in terms of life satisfaction. We therefore used the Satisfaction with Life Scale (SWLS) developed by Diener et al. (1985) for the measurement of life satisfaction as the outcome of a cognitive-judgmental process. The SWLS measures global life satisfaction and consists of five items evaluated on a 7-point scale (1 = *strongly disagree*, 7 = *strongly agree*). Sample items include “In most ways my life is close to my ideal”, “I am satisfied with life”, and “If I could live my life over, I would change almost nothing” with internal consistencies from α = .77 to α = .72 across samples.

## Results

Before examining our hypotheses, the descriptive statistics of each group were computed. Our main hypotheses were tested using multivariate analyses of variance (MANOVA) and path analyses (AMOS, Arbuckle 2009). Therefore, the results are presented in two sections. In the first part, we provide descriptive statistics and group differences in religious identity and acculturation orientations of Turkish youth. Furthermore, we examine the mediational role of acculturation orientations on the relationship between religious identity and adjustment in both groups.

### Group differences in religious identity and acculturation orientations

We examined differences in religious identity and acculturation orientations between Turkish-Bulgarians and Turkish-German groups via a MANOVA with group (2 levels) as independent variable and religious identity and acculturation orientations as dependent variables. Consistent with the assumption that Turkish-Bulgarians belong to a discriminated minority group, they scored higher on host culture adoption (*F*(1, 313) = 84.23, *p* < .001, η^2^ = .213; Turkish-Bulgarian *M* = 3.06, *SD* = .75 vs. Turkish-German *M* = 2.61, *SD* = .79), lower on heritage culture maintenance (*F*(1, 313) = 26.84, *p* < .001, η^2^ = .079; Turkish-Bulgarian *M* = 3.51, *SD* = .91 vs. Turkish-German *M* = 4.22, *SD* = .81) and lower on religious identity (*F*(1, 313) = 51.79, *p* < .001, η^2^ = .143, Turkish-Bulgarian *M* = 3.35, *SD* = .83 vs. Turkish-German *M* = 4.16, *SD* = .87) than Turkish-Germans.

### Acculturation orientations as mediator of the relationship between religious identity and outcomes

Preliminary analyses assessed associations among all study variables for each group via Pearson bivariate correlations (Table 2). Religious identity was significantly and positively related to maintenance, heritage and host culture adjustment and life satisfaction in the Turkish-Bulgarian group. In the Turkish-German group, religious identity was equally positively related to maintenance, heritage culture adjustment and life satisfaction, but negatively related to adoption and host culture adjustment. It was also interesting to observe significant positive relations between acculturation orientations and outcomes (host and heritage) for Turkish-Bulgarians and inverse negative relation for their Turkish-German peers (see Table 2).

**Table 2:** Correlations of study variables per group

	Turkish-Bulgarian						Turkish-German					
	1.	2.	3.	4.	5.	6.	1.	2.	3.	4.	5.	6.
1. Religious identity	–						–					
2. Maintenance	.53**	–					.76**	–				
3. Adoption	.11	.23**	–				−.32**	−.31**	–			
4. Host adjustment	.34**	.31**	.47**	–			−.16*	−.20**	.51**	–		
5. Turkish adjustment	.44**	.62**	.05	.59**	–		.57**	.74**	−.30**	−.01	–	
6. Life satisfaction	.17*	.08	.01	.03	.03	–	.37***	.32***	.01	.07	.29***	–

* *p* < .05

** *p* < .01

**Fig. 2:**
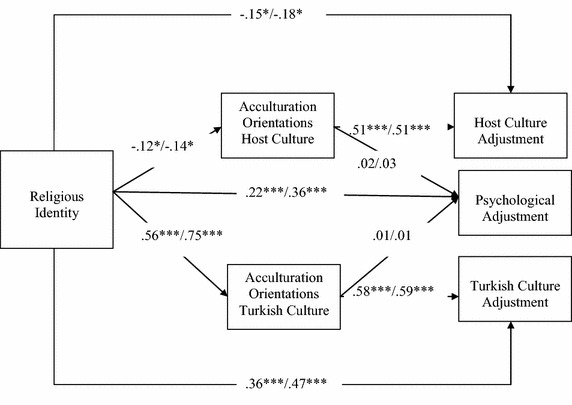
Path model of religious identity, acculturation orientations and socio-cultural adjustment to the Turkish and/or host culture. *Note*: Coefficients refer to the standardized regression coefficients in the structural weights model. First coefficient on the arrow refer to the Turkish-Bulgarian sample and the second coefficient to the Turkish-German sample, χ^2^(19, *N* = 336) = 38.86, *p* < .058, CFI = .968, RMSEA = .058. *p < .05; ***p < .001

In the following, we test our last prediction on the mediational role of acculturation orientations on the relationship between religious identity and adjustment in a path model using AMOS (Arbuckle 2009). Three global fit indices were adopted to interpret the results of the path analyses in relation to the overall model fit: the χ^2^-test, the root mean square error of approximation (RMSEA) and the comparative fit index (CFI). RMSEA ≤ .08, and CFI ≥ .90 were considered as cut-off values for acceptable fit to the data (Browne and Cudeck 1993; Marsh et al. 2005). To test for a possible mediation effect, we first calculated the direct effects of religious identity on host culture adjustment, heritage culture adjustment and psychological adjustment when no mediating variables were introduced, and subsequently compared these effects to direct effects of religious identity on these three outcomes when acculturation orientations to host and heritage culture were introduced as mediating variables. Finally, to examine whether mediation occurred, we tested the indirect effect for significance using bootstrapping. As presented in Table 3, in both samples the indirect effects were significant for Turkish culture adjustment, while the indirect effects were not significant for host culture adjustment and subjective well-being. Hence, the effect of religious identity on Turkish culture adjustment seems to be mediated by adolescents’ maintenance, while there was a significant direct effect on host culture adoption and subjective well-being (see Fig. 2). Results of the multi-group path analysis for testing the applicability and invariance of this model in the two samples provided good fit for the structural weights solution. This indicates that the proposed mediation model was applicable to describe the relationships between religious identity, acculturation orientations and outcomes in both groups, and that strength and direction of the relationships was invariant across groups. In both groups, religious identity was positively related to Turkish maintenance, and Turkish maintenance in turn, was positively related to Turkish culture adjustment. Hence, Turkish maintenance partially mediated the link between religious identity and Turkish adjustment in both groups. Similarly, in both groups, religious identity was negatively related to host culture adoption, which in turn was positively related to host culture adjustment. However, unlike for Turkish culture adjustment, the direct effect between religious identity and host culture adoption was not mediated by acculturation orientation and rather weak in both Turkish samples, suggesting that religious identity played a somewhat less important role for adjustment to the host culture (than for adjustment to the heritage culture). Finally, and as noted above, religious identity also showed a direct positive relationship to life satisfaction in both groups (while acculturation orientations seemed unrelated) (see Fig. 2).

**Table 3:** Comparing direct effects, indirect effects of religious identity and explained variances with and without the mediator variables

	Turkish-Bulgarian (*n* = 160)			Turkish-German (*n* = 153)		
	Psychological adjustment	Host culture adjustment	Turkish culture adjustment	Psychological adjustment	Host culture adjustment	Turkish culture adjustment
Direct effects without mediator variables	.21***	−.20**	.43***	.35***	−.25**	.57***
Direct effects with mediator variables	.22***	−.15*	.36***	.36***	−.18*	.47***
Indirect effects	.01	−.06	.33***	.01	−.07	.46***
*R* ^2^ without mediator variables	5 %	1 %	17 %	12 %	2 %	20 %
*R* ^2^ with mediator variables	5 %	21 %	52 %	13 %	12 %	44 %

* *p* < .05

** *p* < .01

*** *p* < .001

## Discussion

The present study addressed religious identity, acculturation orientations and socio-cultural adjustment and their relations among Turkish minority youth living in two diverse receiving countries, namely Bulgaria and Germany. The first question addressed mean level differences between Turkish-Bulgarian and Turkish-German youth with respect to religious identity and acculturation orientations, and the second question tested the applicability of Arends-Tóth and van de Vijver (2006) acculturation framework in these two samples.

With respect to mean-level differences between groups, we found that Turkish-Bulgarian youth scored lower on religious identity and Turkish culture maintenance and higher on host Bulgarian culture adoption than Turkish-German youth. This is in line with prior research which shows that strong pressure to assimilate leads to a stronger orientation towards the host culture, while it weakens the orientation towards the heritage religion and culture (Dimitrova et al. 2014a, b).

With respect to relationships among religious identity, acculturation orientations and socio-cultural adjustment, the multi-group comparative analyses across groups supported a structural weights solution, thereby indicating that strength and directions of relationships between these components of the acculturation process was invariant in the two samples. Although Turkish youth in Bulgaria and Germany are faced with two very diverse contexts of acculturation, and despite mean-level differences in religious identity and acculturation orientations, the structure and relationships between these variables was invariant: In both groups, religious identity was positively related to Turkish culture maintenance, and negatively related to host culture adoption. Moreover, in both groups maintenance and adoption were strongly and positively related to Turkish culture adjustment and host culture adjustment, respectively (see Fig. 2). Hence, the initially observed differences in correlations between samples (see Table 2) are likely to be random variations, not representing true group differences.

In terms of testing whether acculturation orientations served as mediators between religious identity and adjustment outcomes (Arends-Tóth and van de Vijver 2006), mixed evidence emerged. While a partial mediational model was confirmed for heritage culture adjustment, religious identity exerted direct (non-mediated) effects on life satisfaction and host culture adjustment (together with a main effect of adoption). More specifically, religious identity was positively related to heritage culture adjustment both directly and indirectly through the mediating effect of maintenance; whereas religious identity was directly positively related to life satisfaction and negatively to host culture adjustment.

Nevertheless, it should be noted that our findings emphasize the centrality of religious identity for socio-cultural and psychological adjustment, suggesting that a strong religious identity generally promotes better acculturation outcomes for Turkish youth in both countries. This is in line with prior work confirming the beneficial associations between strong religious identity and acculturation outcomes among ethnic minority adolescents (Abbotts et al. 2004; Junger and Polder 1993; Lopez et al. 2011; Udel et al. 2011; Wallace et al. 2003). Results also show that religious identity seems more relevant for Turkish-culture adjustment than for host culture adjustment. As can be seen in Fig. 2, in both groups religious identity was much strongly associated with Turkish culture maintenance than with host culture adoption, and maintenance, in turn, was much strongly related to Turkish culture adjustment compared to the relationship between adoption and host culture adjustment.

Our results also echo past research confirming the salience of religious identity of Turkish minority groups in the Netherlands and Belgium. For example, studies consistently found that Turks and Moroccans in the Netherlands endorse a very strong Muslim religious identity (Verkuyten 2007; Verkuyten and Yildiz 2009). Members of these groups had the highest possible score on a Muslim identification measure commonly used in social psychological research mirroring some of the items that are used in this study (e.g., ‘My Muslim identity is an important part of my self’, and ‘I identify strongly with Muslims’). Among Muslim minority groups in Belgium, religious belief practices are critically important for the acculturative process, because these are experienced as culturally distant from the host society (Saroglou et al. 2009). Saroglou and Mathijsen (2007) document that upholding one’s religious beliefs reinforce the attachment to one’s culture of origin among Turkish minority groups in Belgium. Turkish and Turkish-Belgian adolescents have been found to be more religious regardless of age, and that religious reaffirmation was strongly related to Turkish heritage culture maintenance, and ethnic identification (Güngör et al. 2012).

Similar as in the research by Verkuyten and Yildiz (2009) conducted in the Netherlands, our findings show that Muslim minority youth with a high religious identification actively distance from the host culture. In our analyses, religious identity showed negative relationships with both host culture adoption and host culture adjustment. It also seems that Turkish culture maintenance and religiosity act in a similar direction such that a strong endorsement of religious identity is consistently accompanied by a strong ethnic identification, and a disengagement from the host culture, as also shown for other Muslim groups in other European contexts as the ones studied here (Saroglou and Mathijsen 2007; Saroglou 2012; Verkuyten 2007). However, it should still be noted, that overall our findings seem to highlight that religious identity plays an *adaptive* role for adolescents` development, as its positive influence on Turkish culture adjustments outweighs its slightly negative impact on host culture adoption in two distinct contexts of acculturation. Hence, it becomes evident that promoting individuals` religious identity is rather beneficial for their adjustment, and that often suspected negative effects for host culture adoption and adjustment (Dimitrova et al. 2014a, b) are comparably weak.

### Limitations and future research

A major limitation of the present research lies in its cross-sectional design. The relationships between religious identity, acculturation orientations and socio-cultural adjustment are merely correlational, and thereby do not allow for causal interpretations. To gain more clarity about the proposed relationships and the roles of acculturation conditions, orientations and outcomes, longitudinal study designs would be needed. Future research might address this issue and provide more certainty about how for instance enhancing adolescents` religious identity will affect their acculturation orientations and socio-cultural adjustment, when being followed over a longer period of time.

A second limitation that should be noted is the use of self-report information only. All variables in our study were assessed through questionnaires by directly asking the adolescents. Such information is likely to be affected by response styles (e.g., social desirability; Paulhus 1991), shared method variance and individuals’ striving for self-consistent answers (Brannick et al. 2010). For more robust conclusions, future studies should also involve other sources of data collection (e.g., parents` or teachers` reports), direct observations or implicit assessments of variables that are related to acculturation orientations and socio-cultural adjustment (e.g., an ecological event sampling study through a smart phone application). Finally, our study only involves two adolescent samples and therefore lacks generalizability to other minority and Turkish  groups in other receiving countries. Future research examining the role of religious identity for socio-cultural adjustment with other groups and Turkish adolescents in other receiving countries would help to clarify whether results are generalizable.

## Conclusions

Religious identity and acculturation orientations affect acculturation outcomes of underrepresented and understudied minority youth with Turkish background in Bulgaria and Germany. We found that Turkish culture maintenance mediated the link between religious identity and adjustment to the Turkish culture for youth in both countries. Moreover, in both groups religious identity was positively related to life satisfaction and slightly negatively related to both host culture (Bulgarian/German) adoption and adjustment. The study provides unique findings, and underlines the centrality of religious identity in two very diverse acculturation contexts, by pointing towards religious identities’ adaptive function; especially in the heritage culture domain. Finally, the results of this study advance relevant implications for research, policy and practice with ethnic minority (Turkish) youth in European contexts. We could provide new empirical evidence on religious identity and acculturation processes among understudied Turkish-Bulgarian youth in Eastern Europe in addition to scholarly work on Turkish-German samples. For all these youth, religious identity and acculturation processes are intertwined within a set of interactions and behaviors regarding how they ought to navigate their cultural heritage and how to respond to cultural challenges of the majority society. How these youth develop religious identity and maintain their heritage culture is crucial to deal with negative effects of these challenges. Policy and practice with Turkish youth should empower their religious identity as well as positive heritage ethnic socialization experiences to enhance life satisfaction and sociocultural adjustment outcomes in their countries of settlement.
